# Targeting the IL-36 receptor with spesolimab mitigates residual inflammation and prevents generalized pustular psoriasis flares

**DOI:** 10.1172/JCI188530

**Published:** 2025-07-01

**Authors:** James G. Krueger, Mrinal K. Sarkar, Mark G. Lebwohl, Akimichi Morita, Kenneth Gordon, Rachael Bogle, Christopher Cole, Anthony Coon, Richard G. Langley, Richard B. Warren, Arash Mostaghimi, Bruce Strober, A. David Burden, Min Zheng, Aaron R. Mangold, Milan J. Anadkat, Jonathan N. Barker, Joseph F. Merola, Lam C. Tsoi, Ming Tang, Kolja Becker, Denis Delic, Christian Thoma, Johann E. Gudjonsson

**Affiliations:** 1 Laboratory for Investigative Dermatology, Rockefeller University, New York City, New York, USA.; 2 University of Michigan, Ann Arbor, Michigan, USA.; 3 Icahn School of Medicine at Mount Sinai, New York, New York, USA.; 4 Nagoya City University, Nagoya, Japan.; 5 Medical College of Wisconsin, Milwaukee, Wisconsin, USA.; 6 Dalhousie University, Halifax, Nova Scotia, Canada.; 7 Dermatology Centre, Northern Care Alliance NHS Foundation Trust & Division of Musculoskeletal and Dermatological Sciences, Manchester Academic Health Science Centre, University of Manchester, Manchester, United Kingdom.; 8 Brigham and Women’s Hospital, Harvard Medical School, Boston, Massachusetts, USA.; 9 Yale University School of Medicine, New Haven, Connecticut, USA and Central Connecticut Dermatology Research, Cromwell, Connecticut, USA.; 10 School of Infection and Immunity, University of Glasgow, Glasgow, United Kingdom.; 11 School of Medicine, Second Affiliated Hospital, Zhejiang University, Hangzhou, Zhejiang, China.; 12 Mayo Clinic, Scottsdale, Arizona, USA.; 13 Washington University School of Medicine, St. Louis, Missouri, USA.; 14 St. John’s Institute of Dermatology, King’s College London, London, United Kingdom.; 15 UT Southwestern Medical Center, Dallas, Texas, USA.; 16 Boehringer Ingelheim (China) Investment Co. Ltd, Shanghai, China.; 17 Boehringer Ingelheim International GmbH, Biberach, Germany.; 18 Division of Rheumatology, University of Michigan and Taubman Medical Research Institute, Ann Arbor, Michigan, USA.

**Keywords:** Dermatology, Inflammation, Neutrophils, Skin

## Abstract

People with generalized pustular psoriasis experience underlying skin inflammation, even in the absence of flares. Spesolimab treatment helps control the inflammation and prevent future flares.

**To the Editor:** Generalized pustular psoriasis (GPP) is a rare, chronic neutrophilic inflammatory disease (1, 2). GPP is associated with mutations in genes related to IL-36 signaling, including *IL36RN* (3), *CARD14* (4), and *AP1S3* (5), which contribute to unregulated activation of the IL-36 inflammatory axis in the epidermis, resulting in a neutrophil-rich inflammatory infiltrate and pustule formation (1–5).

Spesolimab, an anti–IL-36 receptor (IL-36R) monoclonal antibody, is the first targeted and approved therapy for the comprehensive treatment of GPP (1, 2). Spesolimab has been shown to reduce the pathogenic molecular profile associated with GPP flares, showing robust suppression of IL-36 pathway–related signatures and neutrophil mediators after just 1 week of treatment (6). Here, we analyzed the effect of spesolimab on the molecular profile of skin in the absence of flares.

Of 123 patients enrolled in the EFFISAYIL 2 clinical trial (2), 18 patients consented to having (optional) biopsies and participated in a biomarker substudy; 7 provided data for all time points throughout the study period. Figure 1 shows biomarker and gene expression data (*n* = 7). Of these 7 patients, 5 patients did not experience a flare during the 48-week study period; 4 patients received 300 mg spesolimab s.c. every 4 weeks after a 600 mg loading dose, and 1 patient received 150 mg spesolimab s.c. every 12 weeks after a 300 mg loading dose. Two patients, both of whom received placebo, experienced a flare. At randomization, all patients had a GPP Physician Global Assessment score of 1 (almost clear skin), with some patients also having low-grade inflammation visible on clinical assessment (Supplemental Figure 1; supplemental material available online with this article; https://doi.org/10.1172/JCI188530DS1).

Using nonsupervised clustering of the entire EFFISAYIL 2 patient cohort, along with associated disease parameters, all patients in the biomarker substudy were evenly distributed among the larger cohort, suggesting that they are representative of the full study cohort (Supplemental Figure 2). Supplemental Figure 3 shows the overall effect of spesolimab on gene expression and distinct inflammatory pathways during the 48-week treatment period.

To highlight the variability of gene expression, clinical features, and immunohistochemistry, we visualized data to a single-patient view of the 7 patients (Supplemental Figure 1). Six patients had increased expression of a broad range of proinflammatory genes at baseline, some of which have been previously associated with pustular psoriasis and defined as IL-36 response genes (2, 6); this indicates that ongoing subclinical inflammation can be present between flares in the majority of patients. Of note, these 6 patients received systemic nonbiologic treatment for GPP before randomization.

GPP flares were accompanied by increased expression of various proinflammatory genes, which normalized after treatment with i.v. spesolimab within 4 weeks and was maintained at week 48. Of note, most (3 of 4) patients receiving the higher dose of spesolimab (300 mg spesolimab s.c. every 4 weeks after a 600 mg loading dose) had near-complete normalization of their proinflammatory gene expression at week 48 (Figure 1) and did not experience flares. No significant impact of *IL36RN*, *CARD14*, or *AP1S3* mutation status on spesolimab efficacy and transcriptomic changes was observed.

CRISPR/Cas9 was used to knockout *IL36RN* and *AP1S3* in keratinocytes (Supplemental Figure 4) and revealed heightened sensitivity to low-intensity proinflammatory stimuli with either IL-17A or IL-36G. Thus, these mutations may predispose these patients to a more rapid and amplified inflammatory response in the skin.

Importantly, our data demonstrate that patients with a history of GPP flares have subclinical residual inflammatory activity in their skin during periods without flare. We further demonstrate that this inflammatory activity is effectively suppressed with spesolimab s.c. maintenance therapy, decreasing the risk of spontaneous flares, which is consistent with the findings from the EFFISAYIL 2 trial (2).

This study has several limitations. One is the small number of patients, which represent only a small fraction of the overall EFFISAYIL 2 trial. However, the 7 patients were shown to be representative of the overall trial. Another limitation is that only few patients had mutations in genes, such as *IL36RN*, *CARD14*, and *AP1S3*. However, the data demonstrate the effectiveness of anti–IL-36R inhibition with spesolimab regardless of *IL36RN* mutation status.

For detailed methods, information regarding sex as a biological variable, plain language summary, statistics, study approval, author contributions, and acknowledgments, see the supplemental materials.

## Supplementary Material

Supplemental data

Supporting data values

## Figures and Tables

**Figure 1:**
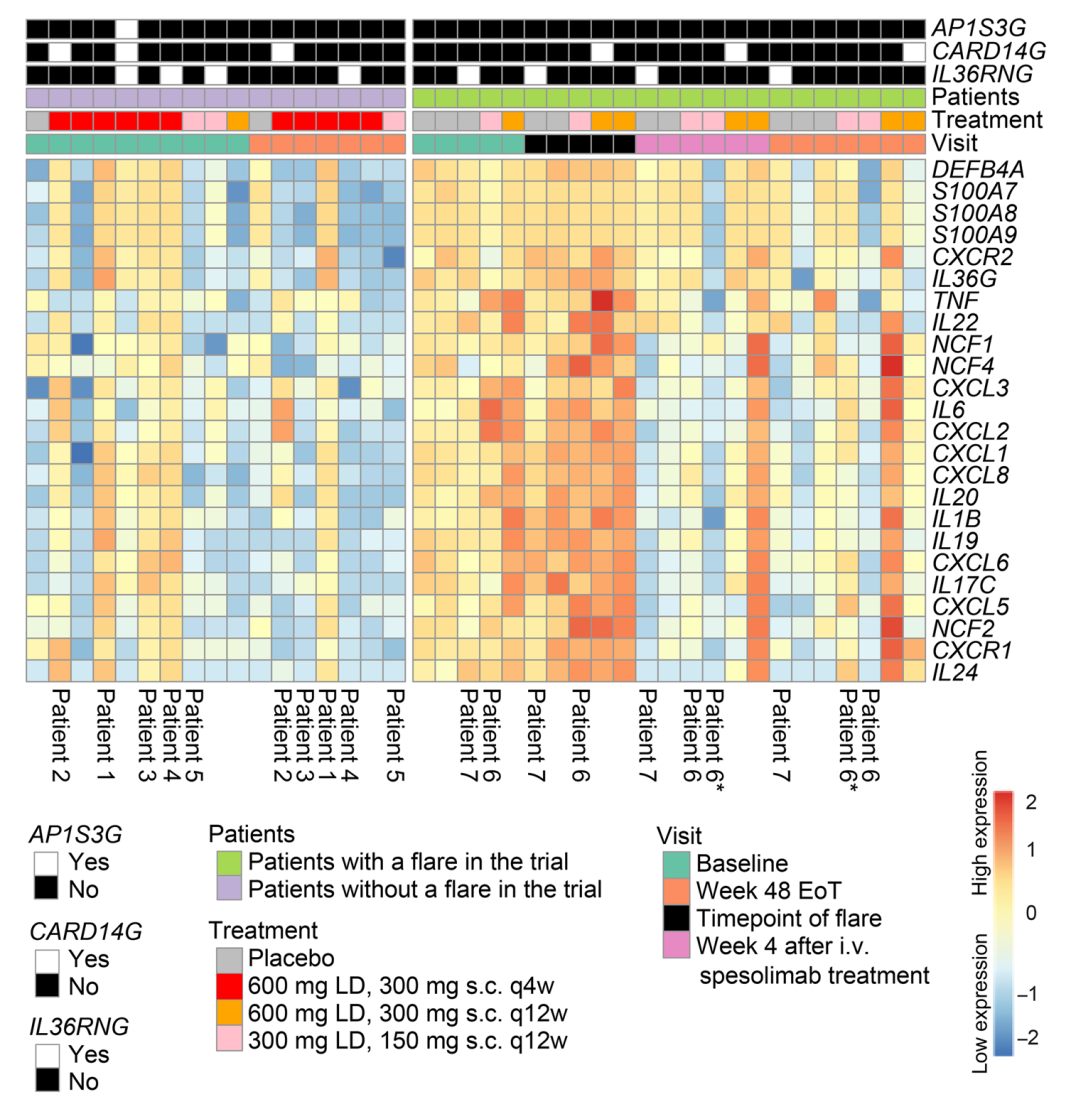
Changes in proinflammatory gene expression during the study period. Alternative biopsies are indicated by asterisks. The graph shows the gene expression of proinflammatory mediators in all 18 patients who participated in the biomarker study at all time points, including flare and postflare treatment and at the end of the study (week 48). Patients who had a complete series of biopsies throughout the studies are shown (Patients 1–7). One patient had more than 1 biopsy (Patient 6). DEFB4A, defensin β 4A; EoT, end of trial; LD, loading dose; NCF, neutrophil cytosolic factor; q12w, every 12 weeks; q4w, every 4 weeks; TNF, tumor necrosis factor.

## Version 1 07/01/2025

In-Press Preview

## Version 2 09/02/2025

Electronic publication
