# Supplementary material for: Targeting the IL-36 receptor with spesolimab mitigates residual inflammation and prevents generalized pustular psoriasis flares
Source: J Clin Invest. 2025 Jul 1;135(17):e188530. doi: 10.1172/JCI188530 (PMC12404765; doi:10.1172/JCI188530)
Supplement: Supplemental data [file jci-135-188530-s132.pdf]

Supplement

Targeting the IL-36 Receptor with Spesolimab Mitigates Residual Inflammation and Prevents Generalized Pustular Psoriasis Flares

Authors: James G. Krueger[1], Mrinal K. Sarkar[2], Mark G. Lebwohl[3], Akimichi Morita[4], Kenneth Gordon[5], Rachael Bogle[2], Christopher Cole[2], Anthony Coon[2], Richard G. Langley[6], Richard B. Warren[7], Arash Mostaghimi[8], Bruce Strober[9], A. David Burden[10], Min Zheng[11], Aaron R. Mangold[12], Milan J. Anadkat[13], Jonathan N. Barker[14], Joseph F. Merola[15], Lam C. Tsoi[2], Ming Tang[16], Kolja Becker[17], Denis Delic[17], Christian Thoma[17], Johann E. Gudjonsson[2,18,19]

1. Laboratory for Investigative Dermatology, Rockefeller University, New York City, NY, USA
2. Department of Dermatology, University of Michigan, Ann Arbor, MI, USA
3. Clinical Therapeutics, Icahn School of Medicine at Mount Sinai, New York City, NY, USA
4. Department of Geriatric and Environmental Dermatology, Nagoya City University, Nagoya, Japan
5. Department of Dermatology, Medical College of Wisconsin, Milwaukee, WI, USA
6. Division of Clinical Dermatology and Cutaneous Science, Dalhousie University, Halifax, NS, Canada
7. Dermatology Centre, Northern Care Alliance NHS Foundation Trust & Division of Musculoskeletal and Dermatological Sciences, Manchester Academic Health Science Centre, University of Manchester, Manchester, UK
8. Department of Dermatology, Brigham and Women's Hospital, Harvard Medical School, Boston, MA, USA
9. Department of Dermatology, Yale University School of Medicine, New Haven, and Central Connecticut Dermatology Research, Cromwell, CT, USA
10. School of Infection and Immunity, University of Glasgow, Glasgow, UK

11. School of Medicine, Second Affiliated Hospital, Zhejiang University, Hangzhou, Zhejiang, China
12. Department of Dermatology, Mayo Clinic, Scottsdale, AZ, USA
13. Division of Dermatology, Washington University School of Medicine, St. Louis, MO, USA
14. St. John's Institute of Dermatology, King's College London, London, UK
15. Department of Dermatology and Department of Medicine, Division of Rheumatology, UT Southwestern Medical Center, Dallas, TX, USA
16. Boehringer Ingelheim (China) Investment Co. Ltd, Shanghai, China
17. Boehringer Ingelheim International GmbH, Biberach, Germany
18. Department of Internal Medicine, Division of Rheumatology, University of Michigan, Ann Arbor, MI, USA
19. Taubman Medical Research Institute, Ann Arbor, MI, USA

Conflict of Interest: JGK has received research support from AbbVie, Amgen, Artax Biopharma, Avillion Life Sciences, Biogen, Boehringer Ingelheim, Bristol Myers Squibb, Eli Lilly, Exicure, Innovaderm, Incyte, Janssen, Kyowa Kirin, Novan, Novartis, Ono Pharma, Pfizer, Provectus, Regeneron, Sudo Biosciences, Takeda, UCB, and Vitae Pharmaceuticals; and has received consulting fees from AbbVie, Allergan, Almirall, Amgen, Artax Biopharma, Aristea, Bausch Health, Biogen Idec, Boehringer Ingelheim, Bristol Myers Squibb, Calliditas, Eli Lilly, Escalier Biosciences, Incyte, Janssen, Kymera Therapeutics, Kyowa Kirin, MC2 Therapeutics, Merck, MoonLake Immunotherapeutics, Novartis, Nuvig Therapeutics, Oruka Therapeutics, Pfizer, Sanofi, Sun Pharmaceutical Industries, Takeda, Target-Derm, UCB, UNION Therapeutics, and Ventyx Biosciences. **MGL** has received research funds from AbbVie, Arcutis Biotherapeutics, Avotres, Boehringer Ingelheim, Cara Therapeutics, Clexio, Dermavant, Eli Lilly, Incyte, Inozyme, Janssen, Pfizer, Sanofi-Regeneron, and UCB; and has acted as a consultant for Aikium, Almirall, AltruBio, Amgen, Apogee, Arcutis Biotherapeutics, AstraZeneca, Atomwise, Avotres, Boehringer Ingelheim, Bristol Myers Squibb, Castle Biosciences, Celltrion, CorEvitas, Dermavant, Dermsquared, Evommune, Facilitation of

International Dermatology Education, Forte Biosciences, Galderma, Genentech, Incyte, LEO Pharma, GoodRx-Mayne, Meiji Seika Pharma, Mindera, Mirum Pharmaceuticals, Oruka Therapeutics, Pfizer, Revolo Biotherapeutics, Sanofi-Regeneron, Seanergy, Strata, Sun Pharmaceutical Industries, Takeda, Trevi Therapeutics, and Verrica. **A Morita** declares receiving research grants and/or consulting fees and/or speaker fees from AbbVie, Boehringer Ingelheim, Eli Lilly, Janssen, Kyowa Kirin, LEO Pharma, Maruho, Sun Pharmaceutical Industries, Taiho Pharmaceutical, Torii Pharmaceutical, and Ushio.

KG reports grant support and consulting fees from AbbVie, Boehringer Ingelheim, Bristol Myers Squibb, Celgene, Eli Lilly, Janssen, Novartis, and UCB; and has received consulting fees from Amgen, Almirall, Dermira, LEO Pharma, Pfizer, and Sun Pharmaceutical Industries. **RGL** has received honoraria as a principal investigator, scientific advisor, or speaker for AbbVie, Amgen, Boehringer Ingelheim, Bristol Myers Squibb, Dermavant, Dermira, Eli Lilly, GSK, Janssen, LEO Pharma, Novartis, Ortho Dermatologics, Pfizer, Sanofi, Sun Pharmaceutical Industries, and UCB. **RBW** declares receiving research grants from AbbVie, Almirall, Amgen, Celgene, Eli Lilly, Janssen-Cilag, LEO Pharma, Medac, Novartis, Pfizer and UCB; and consultancy fees from AbbVie, Almirall, Amgen, Arena, Astellas, Avillion, Boehringer Ingelheim, Bristol Myers Squibb, Celgene, DICE, Eli Lilly, GSK, Janssen, LEO Pharma, Medac, Novartis, Pfizer, Sanofi, Sun Pharmaceutical Industries, UCB, and UNION Therapeutics. **A Mostaghimi** has received consulting fees from AbbVie, ACOM Health, Bioniz, Boehringer Ingelheim, Concert, Digital Diagnostics, Eli Lilly, Equillium, Hims & Hers Health, and Pfizer; equity from ACOM Health, Figure 1, and Hims & Hers Health; licensing/royalties from Concert and Pfizer; and research funding from Aclaris, Concert, Eli Lilly, and Incyte. **BS** is a consultant for and has received honoraria from AbbVie, Alumis, Almirall, Amgen, Apogee, Arcutis, Boehringer Ingelheim, Bristol-Myers-Squibb, Capital One, CorEvitas, Dermavant, Immunovant, Janssen, Leo, Eli Lilly, Maruho, Oruka Therapeutics, Meiji Seika Pharma, Protagonist, Takeda, Novartis, Pfizer, UCB Pharma, RAPT Therapeutics, Regeneron, Sanofi, and UNION Therapeutics. He has stock options in Connect Biopharma and Mindera Health, and has acted as a speaker for AbbVie, Arcutis,

Dermavant, Eli Lilly, Incyte, Janssen, Regeneron, and Sanofi. He is an investigator for and receives consulting fees as Scientific Co-Director of the CorEvitas Psoriasis Registry and receives honoraria as editor-in-chief of the *Journal of Psoriasis and Psoriatic Arthritis*. **ADB** declares paid consulting activities for AbbVie, Almirall, Boehringer Ingelheim, Bristol Myers Squibb, Celgene, Eli Lilly, Janssen, LEO Pharma, Novartis, and UCB. **MZ** has participated in advisory boards and/or acted as an investigator and/or speaker and received grants and/or honoraria from AbbVie, Boehringer Ingelheim, Janssen-Cilag, LEO Pharma China, Novartis, Pfizer, Sun Pharmaceutical Industries, and Xian-Janssen. **ARM** has consulted for Argenx, Boehringer Ingelheim, Bristol Myers Squibb, Clarivate, Eli Lilly, Incyte, Janssen, Kyowa Kirin, LEO Pharma, Momenta, Nuvig, Pfizer, Phelecs BV, PPD, Regeneron, Soligenix, Tourmaline Bio, and UCB; has received grant support from AbbVie, Argenx, Corbus, Eli Lilly, Elorac, Incyte, Janssen, Kyowa Kirin, Merck, MiRagen, Novartis, Palvella, Pfizer, Priovant, Regeneron, and Sun Pharmaceutical Industries. He has received royalties from Adelphi Values and Clarivate; his current patents include Methods and Materials for assessing and treating cutaneous squamous cell carcinoma (PCT/US2023/078902), Use of Oral Jaki in Lichen Planus (PCT/US2024/020149), Topical Ruxolitinib in Lichen Planus (PCT/US2021/053149, 2023-520085, & 21805700.8, respectively), and Methods and Materials for Treating Lichen Planopilaris (Registration Number: 53,103). **MJA** declares paid activities as a current advisor or consultant for Biolinq, Boehringer Ingelheim, Eli Lilly, Novocure, Protagonist Therapeutics, and SpringWorks Therapeutics; has served as a prior consultant or advisor for AbbVie, Amgen, Innovaderm Research, Kintara Pharmaceuticals, Novartis, OnQuality Pharmaceuticals, and UCB; and has served as an investigator for AbbVie, AstraZeneca, Boehringer Ingelheim, Eli Lilly, Incyte, Novartis, Novocure, OnQuality Pharmaceuticals, and Regeneron. **JNB** has received honoraria and/or research grants from AbbVie, Almirall, Amgen, Boehringer Ingelheim, Bristol Myers Squibb, Celgene, Eli Lilly, Janssen, LEO Pharma, Novartis, Samsung, and Sun Pharmaceutical Industries. **JFM** has acted as a consultant and/or investigator at AbbVie, Amgen, Biogen, Boehringer Ingelheim, Bristol Myers Squibb, Dermavant, Eli Lilly, Janssen, LEO Pharma, Novartis, Pfizer,

Regeneron, Sanofi, Sun Pharmaceutical Industries, and UCB. **LCT** has received support from Galderma and Janssen. **MT**, **KB**, **DD**, and **CT** are employees of Boehringer Ingelheim. **JEG** has received research grants from AbbVie, Almirall, Boehringer Ingelheim, Bristol Myers Squibb, Eli Lilly, Janssen, Kyowa Kirin, Merck, Prometheus, and Sun Pharmaceutical Industries; and has served as an advisory board member or consultant for AbbVie, Almirall, AnaptysBio, Boehringer Ingelheim, Bristol Myers Squibb, Eli Lilly, Galderma, Janssen, miRagen, Novartis, Oruka Therapeutics, Renovare Sclerobio, Renova Therapeutics, Sanofi, and Takeda. **MKS, RB, CC** and **AC** report no conflicts of interest.

Funding: This study was funded by Boehringer Ingelheim.

Disclosures: All authors meet the International Committee of Medical Journal Editors (ICMJE) criteria for authorship, take responsibility for the integrity of the work as a whole, and have given their approval for this version of the article to be published. Boehringer Ingelheim was given the opportunity to review the manuscript for medical and scientific accuracy as well as intellectual property considerations. The authors did not receive payment related to the development of the manuscript. Eleanor James, BSc, of Nucleus Global provided editorial and formatting support, which was contracted and funded by Boehringer Ingelheim.

Acknowledgements: This study was supported by Boehringer Ingelheim. JEG and LCT are supported by NIH P30, AR075043. MKS received support from the National Psoriasis Foundation. The authors would like to acknowledge the contribution of David Hall to this manuscript. Additionally, the authors thank Sudha Visvanathan for contributing to the preparation of this manuscript.

Author contributions: JEG, CT and ADB conceived the study. JEG, CT, ADB and MT designed the methodology. MT developed the software. MT, KB, KG and LCT performed the formal analysis. MKS, LCT, RB, CC, AC, KB and DD carried out the investigation. RB and KB curated the data. JEG and CT wrote the original draft. LCT, RB, and JEG were

responsible for the preparation, creation and/or presentation of the published work, specifically visualization/data presentation. JEG had responsibility for the research activity planning and execution. All authors critically reviewed and revised the draft manuscript and approved the final version for publication.

Plain language summary

Generalized pustular psoriasis (GPP) is a rare, chronic skin disease. It causes widespread pus-filled blisters (pustules), skin redness, and pain. These symptoms are caused by inflammation that occurs with GPP. People with GPP experience periods called 'flares', when their symptoms suddenly get worse. Many people with GPP also have ongoing symptoms between flares.

A treatment called spesolimab is approved in many countries to treat GPP flares. In a clinical study called EFFISAYIL® 2, researchers aimed to understand the effects of treating patients who were not having a GPP flare with spesolimab, to see if this could stop flares from happening. In this smaller sub-study of some patients from EFFISAYIL® 2, researchers wanted to understand more about skin inflammation in patients with GPP and if spesolimab could help reduce it. Skin samples were analyzed for seven participants, of whom five received spesolimab and two received placebo (an injection not containing any medication).

Researchers found that between flares, and even when there are no visible skin symptoms, patients with GPP still have inflammation in their skin. The study showed that regular injections of spesolimab were effective in reducing this hidden inflammation.

The study also explored why some people are more likely to have GPP flares. It found that certain genetic changes (mutations) make skin cells extra sensitive to signals that cause inflammation.

Overall, the findings of this sub-study suggest that continuous treatment with spesolimab can reduce underlying inflammation in the skin of people with GPP, even when no active symptoms are present.

Supplementary methodology

Sex as a biological variable

To determine whether participants included in this biomarker sub-study were representative of the overall study cohort, non-supervised clustering of the entire patient cohort from the EFFISAYIL® 2 trial, including biological sex, was performed. The sub-study population was found to be representative of the larger cohort of patients in the EFFISAYIL ® 2 trial; therefore, sex was not considered to be a confounding biological variable in these analyses.

Patients

The study design of the larger EFFISAYIL® 2 trial has previously been described, including study sites and eligibility criteria (1). In short, the study was a multicenter, randomized, placebo-controlled study carried out at 60 hospitals and clinics across 20 countries (1). Study participants were 12 to 75 years old with a documented history of GPP as per the European Rare and Severe Psoriasis Expert Network Criteria (1, 2). A subset of patients from EFFISAYIL® 2 were enrolled in a biomarker sub-study, conducted over 48 weeks.

Skin biopsy procedure

Skin biopsies (5 mm punch biopsies) were taken at baseline and week 48 (the end of the study); in patients who flared, biopsies were also taken at the time of the flare and 4 weeks post-flare.

Immunohistochemistry

Skin biopsies were evaluated for histopathology and immunohistochemistry (IHC) using specific antibodies against the proliferation marker Ki67, lipocalin (LCN)-2, IL-36 receptor expression, and neutrophil elastase as previously described (3), including for CD11c (mouse) B-LY6 clone, BD Pharmingen Cat# 550375, concentration 1:100; Dclamp (mouse), Beckman Coulter Cat # PN IM3448, concentration 1:100; LCN-2 (mouse), Abcam Cat# ab23477, concentration 1:50; S100A7 (Psoriasin) (mouse) 47C1068 clone, Abcam Cat#

13680, concentration 1:1000; Ki-67 (mouse), MIB-1 clone, STA, CRUZ Cat# sc-101861, concentration 1:25; CD3 (mouse), SK7, BD Biosciences Cat# 347340, concentration 1:100; K16 (mouse), 7A4 clone, LS BIO Cat# LS-B14481, concentration 1:1000; IL17c (rabbit), Polyclonal, LS BIO Cat# LS-C406475, concentration 1:300; HBD2 (goat), Polyclonal P161G, Peprotech Cat# 500-P161G, concentration 1:20. The PBS solution was used for washing at 0.1 M for 5 min between each change of reagents. The slides were developed at room temperature. Global histopathologic scores were assigned to each skin biopsy sample as previously described (4, 5). Histologic biomarkers associated with GPP disease, including CD11c dendritic cells, CD3 T cells, and neutrophil elastase-, LCN-2-, and IL-36γ-expressing cells, were assessed in skin biopsies by IHC at baseline and week 48.

RNA isolation and transcriptomic sequencing and analyses

Skin biopsies were collected prior to spesolimab administration at the first visit (baseline; lesional and non-lesional), at time of flare and at week 4 post-flare (lesional), and week 48 (end of study; lesional). Skin biopsies were analyzed using a similar procedure described in EFFISAYIL® 1 (6); 5 mm punch biopsies were obtained to use half for RNA sequencing and half for IHC analysis. RNA was extracted from skin samples with the RNeasy Fibrous Tissue Mini Kit (Qiagen, Valencia, CA, USA), and subsequent global transcriptome-wide RNA sequencing (Illumina Novaseq 6000; Illumina, San Diego, CA, USA) of lesional and non-lesional skin biopsy specimens was performed to characterize molecular responses to spesolimab treatment versus placebo.

Keratinocyte experiments

CRISPR KO keratinocytes were generated by an insertion or deletion of single or multiple nucleotides induced by an sgRNA designed from the beginning (5’ end) of the coding sequence/ORF of the target gene. sgRNA target sequences *IL36A*, *IL36B*, *IL36G*, *IL36RN*, and *IL1RL2*/*IL36R* (IL36AsgRNA1: GAAAATTGACACACCTCAGC; IL36BsgRNA1: TCGAGAATCACGAATAGCAT; IL36GsgRNA1:TATCACATGCAAGTATCCAG;

IL36RNsgRNA1: AATAACCAGCTTCTAGCTGG; IL1RL2sgRNA1: AATGTCCTTGCATCCATCTA) were generated using a web interface for CRISPR design (https://design.synthego.com/). Complementary synthetic oligos flanked with a Bbs-I restriction site for each sgRNA targeting sequence were purchased from Millipore-Sigma. Those complementary oligos (for each gene) were annealed and then ligated into pSpCas9 (BB)-2A-GFP (PX458) (Addgene plasmid # 48138), a CRISPR backbone vector. Ligated plasmids were then cloned into competent *Escherichia coli* (ThermoFisher # C737303), and then sgRNA target sequence insertion was verified using Sanger sequencing. This plasmid was then transfected into an immortalized keratinocyte line (N/TERTs) using the TransfeX transfection kit (ATCC # ACS4005) in the presence of a JAK1/JAK2 inhibitor, baricitinib. GFP-positive single-cell sorting was performed into a 96-well plate using a Flow sorter at the University of Michigan Flow Cytometry Core. Single-cell colonies were grown up to ~50% confluency. Cells were then transferred from 96-well plates into 12-well plates and grown again up to ~50% confluence (higher confluency might lead to the differentiation of keratinocytes). Each clone was divided into two parts; one was used to isolate DNA for genotyping, and another was stored for further analysis after completing genotyping. DNA was extracted, and PCR was performed using corresponding genotyping primers. KO keratinocytes, along with the control KO line, were stimulated with IL-17A (1 ng/ml, 10 ng/mL and 20 ng/mL, R&D Systems # 7955-IL) and IL-36G (1 ng/ml, 5 ng/mL and 10 ng/mL, R&D Systems # 6835-IL) separately. RNAs were isolated from cell cultures using a Qiagen RNeasy plus kit (Cat # 74136). Reversed transcription was performed using a High-Capacity cDNA Transcription kit (ThermoFisher # 4368813). Quantitative PCR was performed on a 7900HT Fast Real-time PCR system (ThermoFisher) with TaqMan Universal PCR Master Mix (ThermoFisher # 4304437) using TaqMan primer.

Statistics

The Partition Around Medoids (PAM) method provided the unsupervised clustering of patients. This clustering was based on various factors, including baseline demographic

characteristics, inflammatory parameters, history of flares, and baseline systemic medications. The clustering of patients and the baseline characteristics represented in Supplementary Figure 2 were derived using the Minkowski distance.

Study approval

The EFFISAYIL® 2 trial was conducted in accordance with the trial protocol, the International Council for Harmonisation Good Clinical Practice guidelines, Regulation No. 536/2014 (EU), the Japanese Good Clinical Practice regulations, and applicable local regulations. The study protocol was approved by the ethics committees of participating institutions and countries. The EFFISAYIL® 2 trial is registered with ClinicalTrials.gov (NCT04399837). All patients provided written informed consent, and confidentiality agreements were in place between authors and Boehringer Ingelheim. Patient consent was obtained for the inclusion of patient photographs in this publication.

Data availability

To ensure independent interpretation of clinical study results and enable authors to fulfil their role and obligations under the ICMJE criteria, Boehringer Ingelheim grants all external authors access to relevant clinical study data. In adherence with the Boehringer Ingelheim Policy on Transparency and Publication of Clinical Study Data, scientific and medical researchers can request access to clinical study data, typically, one year after the approval has been granted by major Regulatory Authorities or after termination of the development program. Researchers should use the https://vivli.org/ link to request access to study data and visit https://www.mystudywindow.com/msw/datasharing for further information. All data generated or analyzed during this study are included in this published article or as supplementary information files. Supporting data values for all graphs (Supplementary Figure 4) can be found in the accompanying XLS file.

Supplementary data

To determine whether these patients were representative of the study cohort, we performed non-supervised clustering of the entire patient cohort from the EFFISAYIL® 2 clinical trial, along with associated disease parameters including baseline systemic medication use, disease duration, age, race, sex, weight/BMI, height, disease severity, skin symptoms (pain, pustular activity), concomitant plaque psoriasis, quality of life, inflammatory activity (C-reactive protein, neutrophils), and mutational status for *IL36RN*, the most common predisposing mutation for GPP (7-9). The seven patients in the biomarker sub-study were evenly distributed amongst the larger cohort regarding these parameters (Supplementary Figure 2A) and amongst three significant, but overlapping, clusters of demographic and clinical disease parameters (Supplementary Figure 2B). Thus, these analyses suggest that the individuals who participated in the biomarker sub-study were representative of the larger cohort of patients in the EFFISAYIL® 2 trial.

Using principal component analysis, we observed that most baseline samples from the 7 patients were separated from the post-spesolimab treatment samples at week 48. Notably, the samples obtained from patients having a GPP flare grouped with the baseline samples (red/green symbols vs. blue symbols; Supplementary Figure 3A). Using a threshold of greater than twofold change and an FDR of <0.05, we observed 1,311 differentially expressed genes; of those, 693 were decreased and 618 were increased in week 48 samples compared with baseline. Notable decreased genes included IL36A (log2[fold change; FC])=-7.18, FDR=2.1×10^{-3}), DEFB4B (log2[FC])=6.05, FDR=2.6×10^{-3}), and S100A7A (log2[FC]=5.74, FDR=7.5×10^{-4}), whereas increased genes included epidermal genes such as ELOVL3 (log2[FC]=3.2, FDR=2.5×10^{-3}), LCE5A (log2[FC]=3.1, FDR=2×10^{-2}), and LORICRIN (log2[FC]=2.9, FDR=1.1×10^{-2}) (Supplementary Figure 3B).

Spesolimab treatment decreased the expression of genes involved in biological processes that were enriched at baseline, including regulation of T-cell responses, cytokine-receptor

activity, leukocyte chemotaxis, and defense response to a bacterium (Supplementary Figure 3C). A heatmap of the expression of various pro-inflammatory cytokines in the cohort at baseline, flare (R-Day 1), and post-treatment is shown in Supplementary Figure 3D. Broad suppression of various pro-inflammatory cytokine signatures was seen post-treatment, with strong suppression of IL-36 responses and suppression of other cytokine signatures, including IL-17A, TNF-α, and IFN-γ (Supplementary Figure 3E).

Six patients showed increased expression of antimicrobial genes human beta-defensin 2 (*DEFB4A*), *S100A7*, *S100A8*, and *S100A9*; neutrophil chemokines *CXCL1*, *CXCL2*, *CXCL5*, and *CXCL8* (*IL-8*); and chemokine receptors *CXCR1* and *CXCR2* at baseline (Supplementary Figure 1). Additionally, gene expression of pro-inflammatory cytokines including *IL1B*, *IL6*, *TNF*, and *IL36G* was increased, as well as *IL-20* family members *IL19*, *IL20*, *IL22*, and *IL24*. IHC was performed to validate the transcriptomic findings, highlighting increased epidermal proliferation, as measured by Ki67, neutrophil infiltration through staining of neutrophil elastase, the neutrophil chemokine LCN-2, and prominent expression of the IL-36 receptor in the epidermis.

To determine whether GPP-associated mutations increase the sensitivity of keratinocytes to subclinical inflammatory stimuli, we generated CRISPR/Cas9 knock-outs for *IL36RN* and *AP1S3* in keratinocytes (Supplementary Figure 4A, B). We focused on the mRNA expression of the neutrophil chemokine *CXCL1* and the pro-inflammatory *IL-36* family member *IL36G*. We observed increased expression of *CXCL1* with doses of *IL-17A* as low as 1 ng/mL, amplified in *IL36RN*-deficient keratinocytes. More robust responses were seen with *IL-36G* stimulation, with the responses in *IL36RN*-deficient keratinocytes about 2–3-fold higher than in control lines (Supplementary Figure 4C). Similarly, *IL36RN*-deficient keratinocytes had higher expression of *IL36G* mRNA with both *IL-17A* and *IL-36G* stimulation, but increased expression of *IL36G* mRNA was only seen in *AP1S3*-deficient keratinocytes with *IL-36G* stimulation but not *IL-17A*, consistent with the convergence of these GPP-associated genes on *IL-36* responses.

References

1. Morita A, Strober B, Burden AD, Choon SE, Anadkat MJ, Marrakchi S, et al. Efficacy and safety of subcutaneous spesolimab for the prevention of generalised pustular psoriasis flares (Effisayil 2): an international, multicentre, randomised, placebo-controlled trial. *Lancet.* 2023;402(10412):1541-51.

2. Navarini AA, Burden AD, Capon F, Mrowietz U, Puig L, Koks S, et al. European consensus statement on phenotypes of pustular psoriasis. *J Eur Acad Dermatol Venereol.* 2017;31(11):1792-9.

3. Baum P, Visvanathan S, Garcet S, Roy J, Schmid R, Bossert S, et al. Pustular psoriasis: Molecular pathways and effects of spesolimab in generalized pustular psoriasis. *J Allergy Clin Immunol.* 2022;149(4):1402-12.

4. Visvanathan S, Baum P, Vinisko R, Schmid R, Flack M, Lalovic B, et al. Psoriatic skin molecular and histopathologic profiles after treatment with risankizumab versus ustekinumab. *J Allergy Clin Immunol.* 2019;143(6):2158-69.

5. Krueger JG, Ferris LK, Menter A, Wagner F, White A, Visvanathan S, et al. Anti-IL-23A mAb BI 655066 for treatment of moderate-to-severe psoriasis: Safety, efficacy, pharmacokinetics, and biomarker results of a single-rising-dose, randomized, double-blind, placebo-controlled trial. *J Allergy Clin Immunol.* 2015;136(1):116-24 e7.

6. Choon SE, Lebwohl MG, Marrakchi S, Burden AD, Tsai T-F, Morita A, et al. Study protocol of the global Effisayil 1 Phase II, multicentre, randomised, double-blind, placebo-controlled trial of spesolimab in patients with generalized pustular psoriasis presenting with an acute flare. *BMJ Open.* 2021;11(3):e043666.

7. Marrakchi S, Guigue P, Renshaw BR, Puel A, Pei XY, Fraitag S, et al. Interleukin-36-receptor antagonist deficiency and generalized pustular psoriasis. *N Engl J Med.* 2011;365(7):620-8.

8. Onoufriadis A, Simpson MA, Pink AE, Di Meglio P, Smith CH, Pullabhatla V, et al. Mutations in IL36RN/IL1F5 are associated with the severe episodic inflammatory skin disease known as generalized pustular psoriasis. *Am J Hum Genet.* 2011;89(3):432-7.

9. Sugiura K, Takemoto A, Yamaguchi M, Takahashi H, Shoda Y, Mitsuma T, et al. The majority of generalized pustular psoriasis without psoriasis vulgaris is caused by deficiency of interleukin-36 receptor antagonist. *J Invest Dermatol.* 2013;133(11):2514-21.

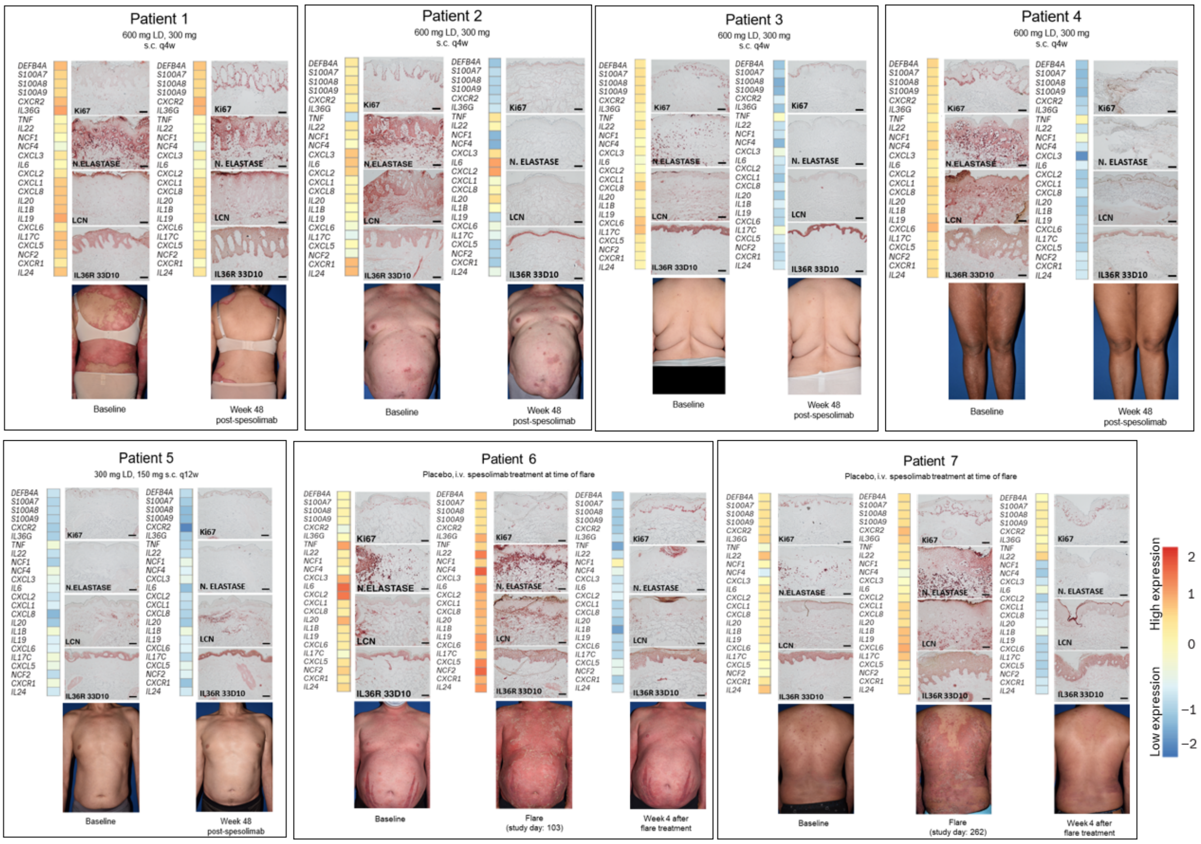

Supplementary Figure 1. Increased inflammatory activity at baseline and reduction at week 48 post-spesolimab in patients enrolled in EFFISAYIL® 2 (patients 1–5), and increased inflammatory activity at baseline, at time of flare, and reduction at week 4 post-spesolimab (patients 6–7). Of the 7 patients enrolled in EFFISAYIL® 2, six showed increased baseline expression of *IL-36* response genes (shown as z-score) along with increased keratinocyte proliferation (shown as Ki67), neutrophil infiltration (neutrophil elastase staining), increased expression of LCN-2, and constitutive expression of the IL-36 receptor in the epidermis. Patients 6 and 7 were randomized to placebo and had increased inflammatory activity at flare onset and reduction 4 weeks after spesolimab 900 mg i.v. flare treatment. Mutation information on *IL36RN*, *CARD14*, and *AP1S3* was as follows: Patient 2 *CARD14* nucleotide exchange: c.599G>A (heterozygous), amino acid exchange: p.Ser200Asn (missense variant); Patient 4 *IL36RN* nucleotide exchange: c.17C>T (heterozygous), amino acid exchange: p.Ala6Val (missense variant); Patient 7 *IL36RN* nucleotide exchange: c.227C>T (homozygous) and c.115+6T>C (homozygous), amino acid exchange: Pro76Leu (missense variant) and splice region variant and intron variant.

Scale bar (IHC panels): 100 µm. DEFB4A, defensin beta 4A; IL, interleukin; i.v., intravenous; LCN, lipocalin; LD, loading dose; NCF, neutrophil cytosolic factor; q12w, every 12 weeks; q4w, every 4 weeks; s.c., subcutaneous; TNF, tumor necrosis factor.

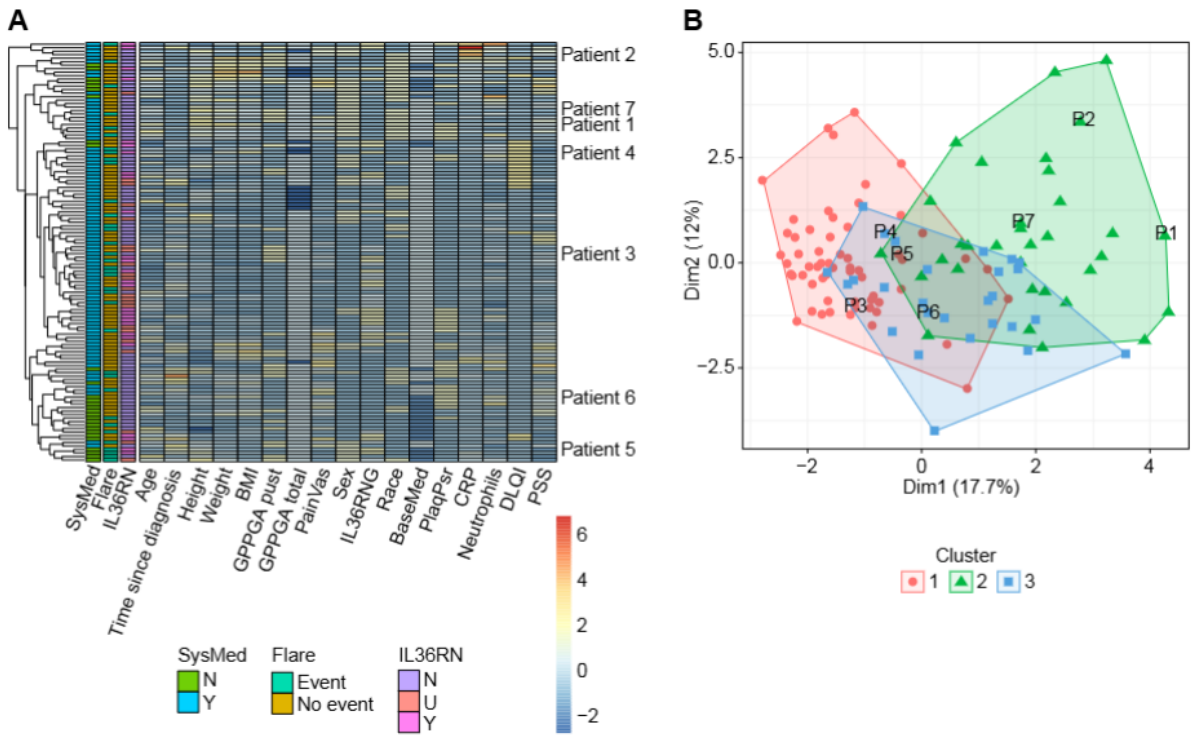

Supplementary Figure 2. Distribution of patients amongst the larger EFFISAYIL® 2 cohort. (A) Heatmap of the study participants in the EFFISAYIL® 2 trial with individual patients in the biomarker sub-study highlighted. The image is displayed as an unsupervised clustering of the patient cohort based on demographic information, including sex and age, mutational status (*IL36RN*), inflammatory parameters (CRP/neutrophils), history of flares and systemic medications. **(B)** The same patient cohort displayed as principal component analysis showing three significant, but overlapping, clusters of demographic and clinical disease parameters and even distribution of the seven study participants amongst the clusters.

Hierarchical clustering was applied in (A), and Partitioning (clustering) Around Medoids (PAM) was applied in (B).

BaseMed, baseline medication; BMI, body mass index; CRP, C-reactive protein; Dim, dimension; DLQI, Dermatology Life Quality Index; GPPGA, Generalized Pustular Psoriasis Physician Global Assessment; IL, interleukin; N, no; PainVas, pain Visual Analogue Scale;

SysMed, systemic medication; PSS, Psoriasis Symptom Scale; pust, pustular psoriasis; U, unknown; Y, yes.

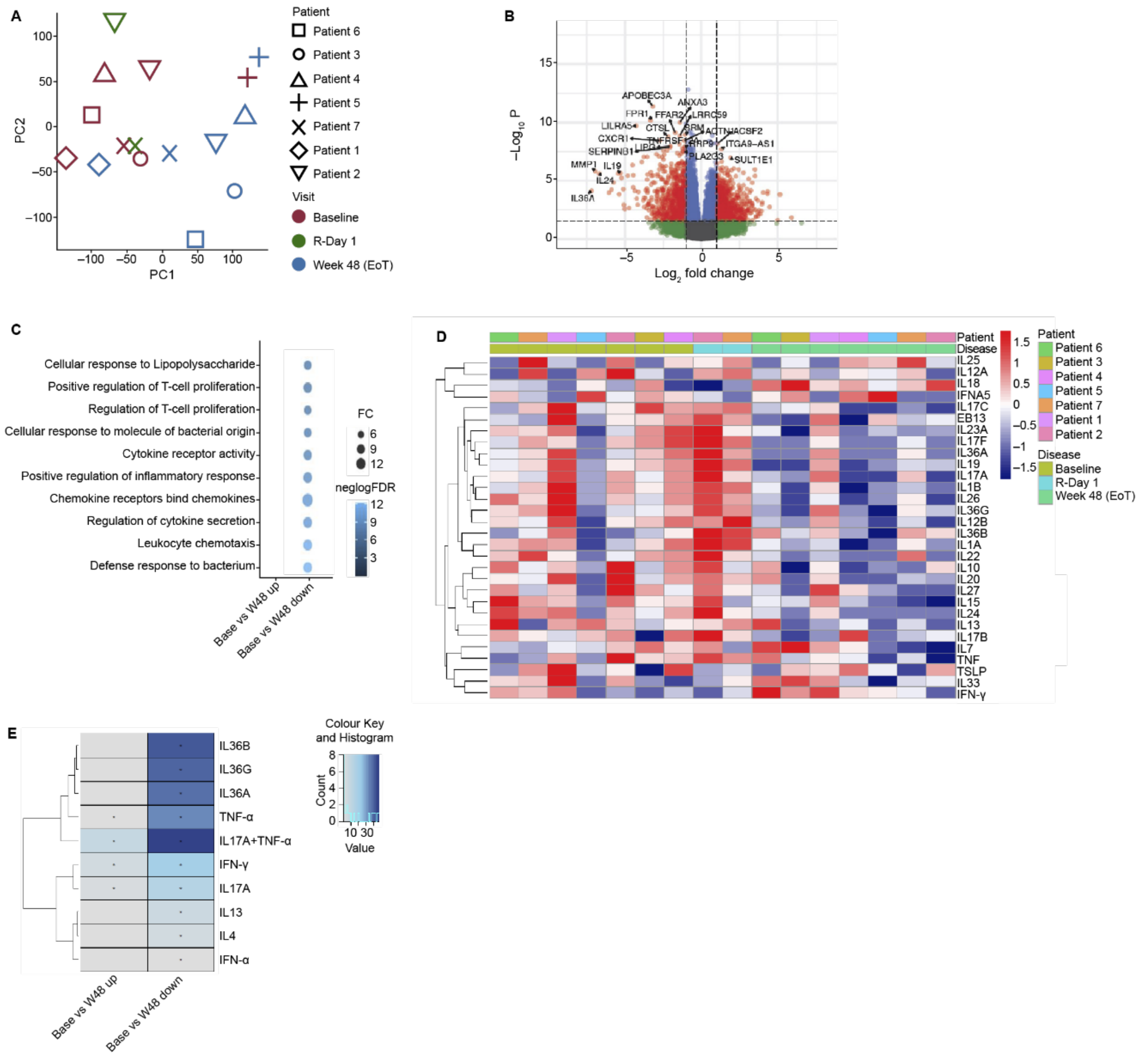

Supplementary Figure 3. Transcriptomic characterization of the EFFISAYIL® 2 biomarker sub-study cohort. (A) Principal component analysis shows overlap of patient samples at baseline but a marked shift at week 48. **(B)** Volcano plot of differentially expressed genes in the data set using a threshold of greater than twofold change and an FDR of <0.05. **(C)** Enriched biological processes were suppressed at week 48 compared with baseline. **(D)** Heatmap of selected cytokine genes across the study cohort and different time points showing shifts towards an overall and marked decrease in cytokine gene mRNA expression at week 48. **(E)** Analysis of cytokine response gene signatures amongst genes decreased at week 48 compared with baseline, showing marked suppression of IL-36 as well as IL-17 and TNF responses.

ACSF2, acyl-CoA synthetase family member 2; ACTN1, actinin alpha 1; ANXA3, annexin A3; APOBEC3A, apolipoprotein B mRNA editing enzyme catalytic subunit 3A; CTSL, cathepsin L; EoT, end of trial; FC, fold change; FFAR2, free fatty acid receptor 2; FPR1, formyl peptide receptor 1; GPP, generalized pustular psoriasis; IL, interleukin; LILRA5, leukocyte immunoglobulin like receptor A5; LIPG, lipase G, endothelial type; LRRC59, leucine rich repeat containing 59; Pat, patient; PC, principal component; PLA2G3, phospholipase A2 group III; R, randomization; RRP9, ribosomal RNA processing 9; SERPINB1, serpin family B member 1; SRM, spermidine synthase; SULT1E1, sulfotransferase family 1E member 1; TNF, tumor necrosis factor; TNFRSF12A, TNF receptor superfamily member 12A; TSLP, thymic stromal lymphopoietin.

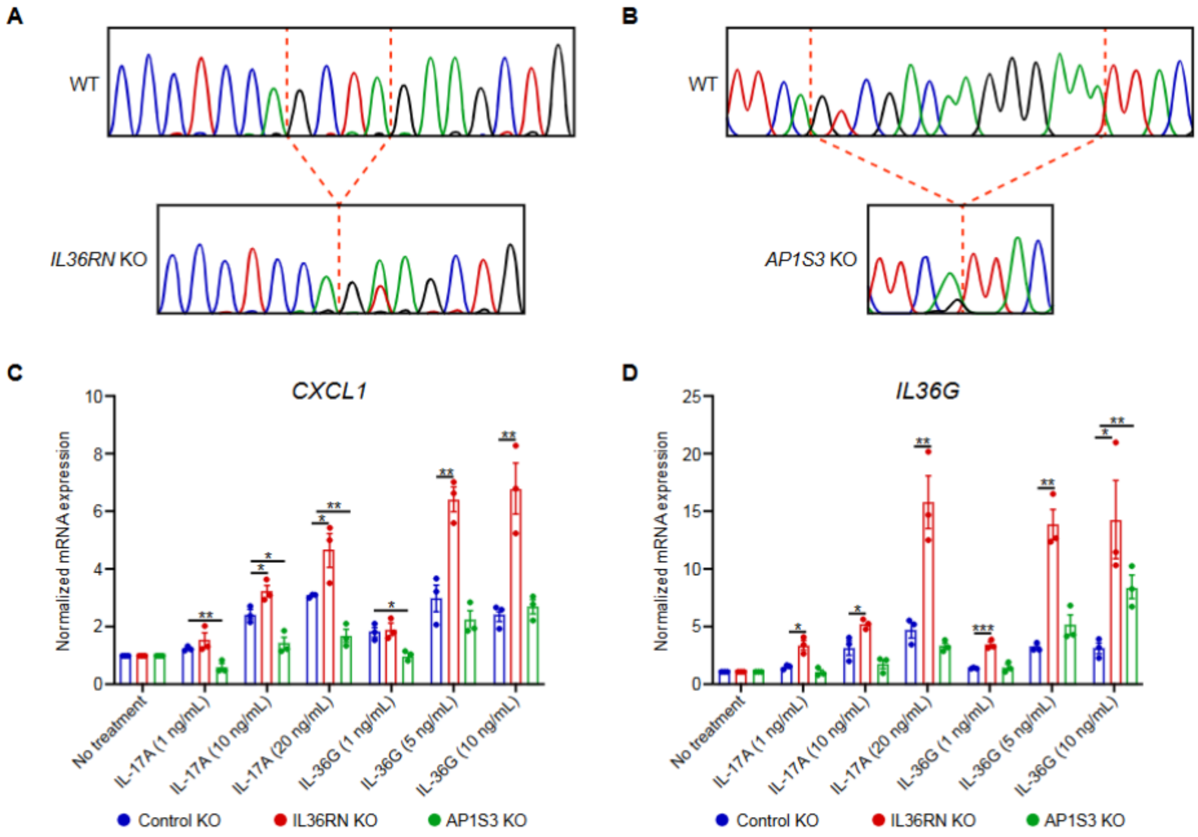

Supplementary Figure 4. GPP-associated mutations heighten sensitivity and amplify keratinocyte responses to inflammatory stimuli. (A) Chromatogram showing a 4-nucleotide deletion causing a frame-shift mutation in the *IL36RN* gene. **(B)** Chromatogram showing a 14-nucleotide deletion causing a frame-shift mutation in the *AP1S3* gene. **(C)** Amplified *CXCL1* mRNA expression in *IL36RN* KO keratinocytes with varying doses of *IL-17* or *IL-36* stimulation. **(D)** Amplified *IL36G* mRNA expression in *IL36RN* KO keratinocytes with *IL-17* and also, separately, *IL-36* stimulation, and amplified mRNA expression of *IL36G* in *AP1S3* KO keratinocytes with only *IL-36G* stimulation. * $p<0.05$, ** $p<0.01$, Student's t-test. n=3 for control KO, IL36RN KO and AP1S3 KO.

GPP, generalized pustular psoriasis; IL, interleukin; KO, knockout; mRNA, messenger ribonucleic acid; WT, wild-type.
